# An Italian Campaign to Promote Anti-doping Culture in High-School Students

**DOI:** 10.3389/fpsyg.2019.00534

**Published:** 2019-03-11

**Authors:** Roberto Codella, Bill Glad, Livio Luzi, Antonio La Torre

**Affiliations:** ^1^Department of Biomedical Sciences for Health, Università degli Studi di Milano, Milan, Italy; ^2^Metabolism Research Center, IRCCS Policlinico San Donato, San Donato Milanese, Italy; ^3^European Athletics, Lausanne, Switzerland; ^4^IRCCS Istituto Ortopedico Galeazzi, Milan, Italy

**Keywords:** doping, anti-doping, WADA, athletes’ behaviors, athlete’s health

## Abstract

Doping poses a threat to sport worldwide. Studies have revealed that, in addition to elite athletes, amateur and recreational sportsmen and sportswomen are making increasing use of performance-enhancing drugs. Worryingly this trend has been documented among young people. Anti-doping efforts seeking to deter elite athletes from doping through detection of the use of prohibited substances are costly and have not been completely effective either at the top-level or the amateur/recreational level. A thoughtful education program, inspired by honesty and respect, might be more effective in shaping attitudes toward doping in young people and protecting their health. For these reasons, the Italian “Lotta al Doping” (*Fight Against Doping*) project sought to cause a cultural shift in young people by taking anti-doping seminars to high schools. In the 2017–2018 school year we reached more than 20,800 students from 157 high schools through 202 seminars. Before and after the seminars, we administrated anonymous, voluntarily completed surveys with a set of questions (items = 15), taken from the WADA-Play-True-Quiz. Upon completion of the 2-h seminar, the majority of the answers given by the students resulted correct (13 out of 15 items, *p* < 0.000001, McNemar) confirming the value of the initiative. This project stands out as promising in the doping prevention process at the youth and amateur levels.

## Introduction

The phenomenon of doping in sport is not restricted to a small number of elite performers (Wanjek et al., 2007). There are grave concerns that it has expanded into the amateur and recreational levels of sport (Barkoukis et al., 2016; Henning and Dimeo, 2018). Worryingly, the use of substances prohibited under the World Anti-Doping Code (WADA, 2015) is being seen more and more among young people in recreational sport (Backhouse et al., 2009) and, thus, it has become both a societal problem and a public health issue (Backhouse, 2014).

An extensive body of literature reports that a small but significant number (∼3–12%) of adolescent sportsmen have used anabolic-androgenic steroids at some point (Yesalis and Bahrke, 2000; Calfee and Fadale, 2006; Bloodworth and McNamee, 2010). In Italy, in 2014, doping control tests were performed on 1,427 young sportsmen and amateur sportsmen: 4% of them were found positive and 73% said they were taking pharmaceuticals and/or health care products (WADA, 2015).

In another study, nearly 7% of 3,400 Italian high school adolescents reported they had used legal “Performance and Appearance Enhancing Substance, PAES” in the previous three months (Mallia et al., 2013). Student athletes who reported having used legal PAES supplements in the previous three months were ten times more likely to have also used illegal substances than those who did not report having used legal supplements (Mallia et al., 2013). The results of this study supported the “gateway hypothesis,” according to which legal PAES use could represent a “gateway” to doping (Lucidi et al., 2008, 2017).

Adolescents’ doping intentions are influenced by doping-specific belief systems (e.g., stronger attitudes in favor of doping use), accounting for 47% of their variance (Zelli et al., 2010). Along with intentions and attitudes, moral disengagement contributes to a greater use of doping substances (Petroczi, 2003; Lucidi et al., 2008). Determinants of intentions appeared to be stable across gender and types of involvement in sport, and they can be predicted by the “Theory of Planned Behavior, (TPB)” (Lucidi et al., 2004, 2017).

In general, sport authorities have identified doping as a threat to the integrity of their sports, to their popularity and sources of income and, of course, to the health and safety of athletes. The most visible responses have been banning an ever-increasing list of substances, conducting doping control tests and sanctioning competitors found to have violated anti-doping regulations through a system overseen and driven by the World Ant-Doping Agency (WADA).

This detection-deterrence approach is expensive (Backhouse, 2014) and it is only practical to test a relatively small percentage of high-level competitors in any time period, even if the targeting of testing has become more sophisticated and intelligence-driven in recent years. At best, this approach has only been partially successful, as doping cases continue make the headlines, and there is a feeling that it cannot win the battle on its own (Kayser and Smith, 2008). Focusing solely on athletes’ personalities and treating doping as a deviant behavior of the few is misleading (Petróczi, 2007) and might represent a limited view of the challenge and what needs to be done. Moreover, detection-deterrence does not address the problems in recreational sport or among adolescent athletes (Backhouse, 2014).

A second response has been anti-doping education, which can be targeted at both high-performance and recreational athletes. Various anti-doping education programs have relied on a “scare-based” approach (Goldberg et al., 1991a,b), ethics-based education (Elbe and Brand, 2016) and knowledge-based approaches (Goldberg et al., 1996; Goldberg and Elliot, 2005; Elliot et al., 2008) stemming from cognitive research and TPB. Some of these have been revealed to be inefficient (Hauw, 2017) and others need to be upgraded (Backhouse et al., 2009; Backhouse, 2014). From a broader perspective, doping research relies on several social-cognitive groundings (Bandura, 1991), including attitudes, subjective norms (Barkoukis et al., 2013), social and interpersonal experiences (Wiefferink et al., 2007), one’s efficacy beliefs and confidence (Lucidi et al., 2004, 2008).

Interestingly, some anti-doping education programs have targeted perceptions of sports values, social norms and attitudes toward PAES use in sport (Barkoukis et al., 2016). A media literacy intervention has been convincingly used in high-school students (Lucidi et al., 2017).

Our national project, “Lotta al Doping” (*Fight against Doping*), emerged from a recognition that the detection-deterrence approach focused on top-level athletes was not working in Italy and, with regard to young people, may have been counterproductive as it contributes to an association of doping with sporting “heroes” promoted by the media and popular culture as role models for young people.

The project has been an attempt to build on the experience and best practice of previous anti-doping education programs targeting young people (both athletes and non-athletes). Our aim was to test a means of sensitizing significant numbers of adolescent students throughout the country and increasing their knowledge about doping and legitimacy in the hope that this would help to shape their belief systems and social attitudes. At the same time we wanted to promote a culture of health, respect for laws and the rules of sport and collaboration between the young and adults in the community.

The educational content for “Lotta al Doping” was developed to ensure the participants engaged with the domain of doping, received accurate information about the spectrum of issues associated with it and openly addressed the moral and ethical risks of certain attitudes with their peers.

## Materials and Methods

### Design

The project design was based on standardized 2-h seminars for students (age: 15–18 years) delivered in Italian high schools during the school year 2017–2018 by a cadre of expert leaders (*n* = 31).

The expert leaders were professionals (track & field coaches, sport scientists, sport psychologists, physicians) who normally operated in scenarios potentially exposed to the doping phenomenon (high-risk groups). They each received a 3-h standardized training that consisted of an anti-doping-culture kit dealing that covered:

- What is doping?
- The World Anti-Doping Agency (WADA) prohibited substances list.
- Effects on health and behaviors induced by performance-enhancing drugs.
- Doping diffusion: fitness club, internet, education sites, amateur sports fields.
- Doping legislation: national and WADA.
- Doping criminality.
- Ethical and health information intended to form negative attitudes toward doping (moral identity, moral disengagement, growing crisis of legitimacy, anticipated guilt).

After their training, the experts visited schools throughout the country to deliver the seminars in which they covered the above listed topics with an emphasis on doping-related medical aspects (detrimental effects on health), psychological aspects and athletic coaching. The delivery of the seminar included a PowerPoint presentation, scenario analysis, problem-solving, and small group discussions.

Prior to the seminars, the participating students were asked, but not required, to complete an anonymous online questionnaire using a QR-code captured by personal smartphones. The questionnaire was a selection of 15 questions taken from *WADA Play True Quiz* (WADA Play True Quiz, 2018), as authorized by WADA. These questions were previously validated in a WADA-supported project (Manfredini et al., 2010) within a large and differentiated population, including non-athlete- and athlete adolescents (Lamberti et al., 2017).

A team of researchers (FIDAL, University of Milan), led by the principal investigator, identified domains of interest to be covered by the questionnaire (“anti-doping rules,” “legitimacy,” and “nutritional supplements”) and ensured that these domains were covered in the seminar.

Upon completion of the seminars, the students were asked, but not required, to complete the same questionnaire again, and after that they received proper explanations for the corrected answers, as provided by WADA (WADA Play True Quiz, 2018).

The McNemar test was used to compare the number of students giving correct answers between the pre- and post-seminar groups. The internal consistency was evaluated for all items and each corresponding domain using Cronbach’s alpha.

### Ethics Statement

The project “Lotta al Doping” was funded by the Italian Federation of Athletics (FIDAL) and approved by the Italian National Olympic Committee (CONI) and the Italian Ministry of Universities and Research (MIUR). Each of these public bodies has its own ethical board, which approves projects to be conducted on a national level in accordance with nation regulations and guidelines. This project had also the approval of the national anti-doping organization (NADO Italia), which is accredited by the WADA for running any campaign, for lab-testing and for fulfilling any requirement related to ethics and anti-doping regulations.

A letter from MIUR was sent to all the participating high school directors informing them about the aims of the project and its contents. Each director, together with the school’s institutional board (which includes, amongst the others, parental representatives), reviewed the project thoroughly and gave formal consent. Finally, parents or legal guardians gave written informed consent for the participation of all enrolled participants.

No identifiable data were gathered. Subject anonymity was strictly guaranteed since surveys were anonymously released via smartphone. No identifying information – of any sort – was requested.

## Results

**FIGURE 1:**
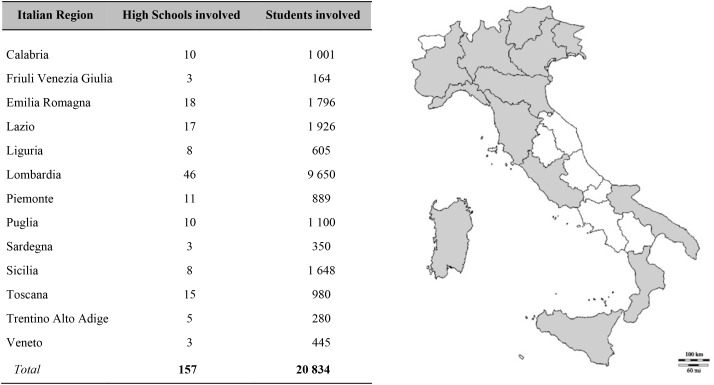
Italian regions (table and map), high schools, and students reached by the “Lotta al Doping” project, during the school year 2017–2018.

The “Lotta al Doping” project reached 157 Italian high schools and a total of 202 seminars were delivered, involving 20,834 students (Figure 1). A total of 3,921 pre-seminar questionnaires were filled in, and 1,544 matched questionnaires were compiled at the end of the seminars.

In the comparison between pre- and the post-seminar responses, students answered correctly, in a significant percentage, in 13 out of 15 items at the end of the seminar (*p* < 0.000001, McNemar test, Table 1).

**Table 1:** Correct answers given by the students to the 15 questions selected from the *WADA Play True Quiz*, before and after the seminars.

	PRE-seminar (%)	POST-seminar (%)	McNemar *p*-value
(1) I am ultimately responsible for what I swallow, inject or apply to my body [Y]	72.9	86.0	<0.000001
(2) WADA stands for: world anti-doping administration, world anti-doping agency? [world-antidoping-agency]	50.4	89.6	<0.000001
(3) Is “unlimited” the maximum number of times an athlete can be tested each year? [Y]	50.0	77.3	<0.000001
(4) If a nutritional supplement is bought from a pharmacy (over-the-counter), it is definitely permitted in sport. [N]	50.0	79.1	<0.000001
(5) When I am sick, I can be excused for taking ANY medicine to help me get well? [N]	50.9	72.2	<0.000001
(6) A coach or doctor assisting or encouraging an athlete to take prohibited substances can be sanctioned if that athlete tests positive? [Y]	67.9	76.2	<0.000001
(7) An athlete can refuse to submit to doping control if he/she is too busy? [N]	73.7	84.2	<0.000001
(8) It is always okay to accept medication from someone you trust, even if you don’t know what the medication contains. [N]	85.9	91.8	<0.000001
(9) The person who receives my sample at the laboratory knows who I am. [N]	50.0	71.4	<0.000001
(10) I can be drug tested during a competition, even if I didn’t compete. [Y]	50.0	66.2	<0.000001
(11) If I am banned in my sport, I can compete in another sport. [N]	70.0	82.7	<0.000001
(12) Can I be found to have committed an anti-doping rule violation (ADRV) if I consume a supplement that is contaminated with a prohibited substance? [Y]	57.8	79.1	<0.000001
(13) A positive test is the only way an athlete can be sanctioned. [N]	50.0	55.9	<0.000001
(14) If I am on particular diet because of a strenuous training, can I assume supplements to improve my performance? [N]	50.0	52.1	0.098086
(15) If I know a supplement has been through a quality control process, I can be guaranteed that it does not contain any substances on the prohibited list. [N]	50.0	50.1	0.401005


*[Y], yes-true; [N], no-false.*

The internal consistency of overall items was excellent (α = 0.962), with high value for “anti-doping rules” domain (α = 0.929), a good value for “legitimacy” (α = 0.850) and “nutrition/diet/supplements” domain (α = 0.880).

## Discussion

According to our preliminary analysis, the intervention was efficacious increasing the level of knowledge about the selected domains.

The levels of participation and interest observed by the expert leaders during the “Lotta al Doping” project 2017–2018 confirm the satisfactory fit of the model across the country. The high school context in which it was delivered was appropriate from both an organizational point of view (we could engage large numbers of participants with minimal promotion) and the fact that it was a safe and familiar setting for the students. Importantly, the project showed that young people desire to be informed on this issue.

This study had several limitations:

(1) In order to maintain anonymity as much as possible, personal information (gender, geographical area) were not collected and therefore responses could not be separated accordingly.
(2) The lack of an actual control group was a general weakness.
(3) A relatively small set of domains was covered by the questionnaires.
(4) The questionnaires were filled-in optionally, and not systematically. This explains the high attrition rate. This convenience sampling procedure may certainly introduce a bias, however, it allowed us to enact an expanded survey while meeting the strict requirements imposed by the participating school institutions.

We are committed to refining the present project by integrating multi-level educational strategies (and quantifying the outcomes) in order to magnify their impact on the anti-doping culture. In fact, alternative approaches should be implemented to augment both young adolescents’ (either athlete- or non-) and health professionals’ knowledge on how improving performance up to or above the level of competitors. Future developments could include multi-structured educational programs to stimulate anti-doping awareness in young people. For example, e-learning platforms like European Athletics’ “I Run Clean^TM^,” which is designed to ensure athletes are exposed to up-to-date information and salutary decision-making could be combined with seminars. This would bring together the online learning that modern young people are used to and a more social setting. It could also be part of a concept to bring anti-doping education initiatives beyond national borders so that doping prevention and fair sport may become two global models to which every athlete and pupil can aspire.

## Conclusion

Clandestine, non-medically supervised use of performance enhancing drugs in the adolescents and general population represents a substantial and increasing public health and social burden. The detection-deterrence approach aimed at elite athletes has not been effective and we need to question current policies and assess alternatives, which should include improved anti-doping education in a variety of settings.

The “Lotta al Doping” was a qualified success and represents a promising step in the effort to prevent doping at the youth and amateur levels.

## Author Contributions

All authors were responsible for drafting the manuscript and revising it critically for valuable intellectual content. All authors approved the final version of the manuscript to be published.

## Conflict of Interest Statement

The authors declare that the research was conducted in the absence of any commercial or financial relationships that could be construed as a potential conflict of interest.

## Abbreviations

FIDAL: italian federation of athletics
MIUR: italian ministry of university and research
NADO: italian national anti-doping organization
PAES: performance and appearance enhancing substances
TPB: theory of planned behavior
WADA: world anti-doping agency
